# Supplementary material for: Dyslipidemia in Children Treated with a BRAF Inhibitor for Low-Grade Gliomas: A New Side Effect?
Source: Cancers (Basel). 2022 May 29;14(11):2693. doi: 10.3390/cancers14112693 (PMC9179293; doi:10.3390/cancers14112693)
Supplement: Supplementary file 1 [file cancers-14-02693-s001.zip › cancers-1716221-supplementary.pdf]

Supplementary Table S1. Detailed demographic and clinical features of the patients treated with vemurafenib.

Pz. N°	Sex	Age at diagnosis	Age at the start of vemurafenib	Tumor site	Tumor Histology	Previous treatment (chemo-radiotherapy)
**1**	F	0,3	4,9	hypothalamic /chiasmatic	ganglioglioma	4 lines of chemotherapy
**2**	F	1,0	2,8	hypothalamic /chiasmatic	ganglioglioma	2 lines of chemotherapy
**3**	M	3,9	9,3	hypothalamic /chiasmatic	ganglioglioma	3 lines of chemotherapy + RT
**4**	F	11,2	11,3	basal ganglia	ganglioglioma	only surgery
**5**	M	13,8	18,8	spinal cord	pilocytic astrocytoma	1 line of chemotherapy
**6**	M	2,8	3,5	hypothalamic /chiasmatic	pilocytic astrocytoma	1 line of chemotherapy

Supplementary Table S2. Lipid levels before and after switch of treatment from vemurafenib to dabrafenib and trametinib.

Lipid category	+1 month Vemur.	+3 months Vemur.	+6 months Vemur.	+12 months Vemur.	+24 months Vemur.	+1 month Dab./Tra.	+3 months Dab./Tra.	+6 months Dab./Tra.
**Total cholesterol**	206	225	191	215	182	135	163	165
**LDL cholesterol**	94	108	70	87	72	50	71	71
**HDL cholesterol**	94	109	119	129	108	80	96	88
**Triglycerides**	78	77	75	52	34	66	68	59

Supplementary Table S3. Fasting blood glucose, biochemical liver and kidney function tests before and during vemurafenib.

Category		-1 month Vemur.	+1 month Vemur.	+3 months Vemur.	+6 months Vemur.	+12 months Vemur.	Last follow up
**Fasting blood glucose (mg/dL)**	Mean ± SD<br>Median (range)	90.5 ± 4.3<br>91 (83 – 95)	86 ± 8.6<br>85 (77 – 99)	85.6 ± 4.5<br>87 (80 – 91)	86.5 ± 5.9<br>87 (80 – 93)	86.8 ± 10.5<br>89 (73 – 100)	93 ± 4.5<br>93 (87 – 99)
**Aspartate aminotransferase (U/L)**	Mean + SD<br>Median (range)	20.2 ± 4.0<br>21 (15 – 25)	29.2 ± 12.4<br>25 (17 – 50)	24.4 ± 9.6<br>20 (15 – 39)	18.9 ± 3.4<br>19 (14 – 24)	21.2 ± 7.1<br>19 (14 – 33)	23.5 ± 6.8<br>21 (18 – 36)
**Alanine aminotransferase (U/L)**	Mean + SD<br>Median (range)	16.3 ± 4.5<br>16 (13 – 25)	32.6 ± 14.2<br>38 (17 – 48)	28.4 ± 14.2<br>23 (11 – 44)	16.5 ± 5.3<br>16 (9 – 23)	15.3 ± 3.3<br>16.5 (10 – 19)	19.2 ± 6.7<br>19 (11 – 30)

**Creatinine**	Mean + SD<br>Median (range)	0.4 ± 0.2<br>0.2 (0.2 – 0.7)	0.6 ± 0.3<br>0.3 (0.3 – 0.9)	0.6 ± 0.3<br>0.3 (0.3 – 1.0)	0.6 ± 0.3<br>0.4 (0.4 – 1.0)	0.6 ± 0.2<br>0.5 (0.5 – 0.9)	0.6 ± 0.1<br>0.5 (0.4 – 0.8)
